# Optimization of Tensile Strength and Cost-Effectiveness of Polyethylene Terephthalate Glycol in Fused Deposition Modeling Using the Taguchi Method and Analysis of Variance

**DOI:** 10.3390/polym16223133

**Published:** 2024-11-10

**Authors:** Dyi-Cheng Chen, Quan-De Zheng, Chih-Hao Chen

**Affiliations:** Department of Industrial Education and Technology, National Changhua University of Education, Changhua 500, Taiwan; m1131019@gm.ncue.edu.tw (Q.-D.Z.); vvtitwo@gmail.com (C.-H.C.)

**Keywords:** fused deposition modeling, polyethylene terephthalate glycol, tensile strength, Taguchi method, ANOVA

## Abstract

This paper investigates the optimization of tensile strength, tensile strength per unit weight, and tensile strength per unit time of polyethylene terephthalate glycol (PETG) material in fused deposition modeling (FDM) technology using the Taguchi method and analysis of variance (ANOVA). Unlike previous studies that typically focused on optimizing a single mechanical property, our research offers a multi-dimensional evaluation by simultaneously optimizing three critical quality characteristics: tensile strength, tensile strength per unit weight, and tensile strength per unit time. This comprehensive approach provides a broader perspective on both the mechanical performance and production efficiency, contributing new insights into the optimization of PETG in FDM. The Taguchi method ($L_{16}\;4^{5}$) was designed and executed, with the layer height, infill density, print temperature, print speed, and infill line direction as the control factors. Sixteen tensile tests were conducted, and ANOVA was employed to identify the main influencing factors for each quality characteristic. For the tensile strength, the infill density was found to have the greatest impact (48.45%), while the print temperature had the least impact (0.78%). The optimal parameter combination reduced the quality loss to 31.28% and standard deviation to 55.93%. For tensile strength per unit weight, the infill line direction had the greatest impact (87.22%), whereas the print temperature had the least impact (0.77%). The optimal parameter combination reduced the quality loss to 54.09% and standard deviation to 73.54%. Regarding the tensile strength per unit time, the layer height had the greatest impact (82.12%), while the print temperature had the least impact (0.08%). The optimal parameter combination reduced the quality loss to 10.81% and standard deviation to 32.87%.

## 1. Introduction

3D printing technology plays a crucial role in various industrial applications by enabling the manufacture of lightweight and geometrically complex parts [1]. As 3D printing technology has advanced, several methods have been developed, including fused deposition modeling (FDM), stereolithography, and laser powder sintering molding [2]. Among these, fused depsosition modeling is extensively used in manufacturing, prototype design, education, and medicine [3,4]. This technology offers several advantages and disadvantages, which are summarized below to clarify the research motivation. 3D printing technology provides numerous benefits. For instance, flexibility in producing complex structures is essential for innovative designs and technological advancements [5]. It facilitates the rapid production of prototypes without needing large inventories, thus saving space and costs while significantly shortening the product development cycle [6]. Additionally, it can produce strong and lightweight parts for the automobile and aerospace industries, enhancing their application value [7]. In the medical field, 3D printing technology is highly advanced; it can produce oral pharmaceuticals and promote the development of medical technology, positively impacting human health [8].

However, 3D printing technology also has limitations. First, material selection is currently limited to plastics and metals, significantly limiting the range of applications [9]. Moreover, the printing space for general models is small, necessitating larger parts to be printed in sections and assembled. This process can lead to tolerance stacking and reduced accuracy [10]. Post-processing, such as cleaning, is often required after printing, which increases manufacturing costs and time [11]. Additionally, scaling up production does not substantially decrease the unit cost of 3D printing, impacting the economic benefits of mass production [12]. The layer-by-layer printing process can also result in delamination under stress, leading to structural defects [13,14]. These limitations constrain the application and development of 3D printing to some extent.

In summary, this paper addresses areas where FDM can be improved despite its advantages, such as low cost and ease of operation. There is considerable potential for enhancing printing quality and the mechanical performance of the machinery. Additionally, the limited range of optional materials restricts the understanding of the properties of non-standard printing materials. To address these issues, this paper employs the Taguchi method and ANOVA to investigate the optimization of tensile strength and unit cost for polyethylene terephthalate glycol (PETG) in FDM. The aim is to advance the application and development of FDM technology and PETG material in the 3D printing field.

## 2. Literature

In 3D printing technology, the Taguchi method and FDM are two essential aspects that are frequently studied and applied. The Taguchi method is an experimental design method that optimizes process parameters to enhance product quality and performance. FDM, a prevalent technique in 3D printing, uses printing process parameters that significantly affect the properties of printed products [15]. Agrawal et al. [16] used concentric circles to fill patterns with an infill density of 80% and a layer height of 100 μm. Their results showed that the tensile strength increased by 123% and 115% compared with the line and triangular patterns, respectively, and the impact strength increased by 168% and 80%, respectively. Ahmad et al. [17] identified that infill line direction had the greatest impact on tensile strength, Young’s modulus, and flexural strength. Fountas et al. [18] revealed the complex relationship between the FDM parameters and tensile strength by developing a statistically validated regression model that thoroughly explained the tensile strength variations induced by the FDM parameters and their nonlinear effects. Kam et al. [19] demonstrated that infill structure, infill rate, and model placement angle directly influenced tensile strength and impact strength, with the difference between experimental results and the prediction model not exceeding 1.8%. They determined that the optimal parameters for tensile strength were a layer height of 0.25 mm, an infill rate of 50%, a linear structure, and a print temperature of 250 °C. Korkut and Hakan [20] found that infill overlap area and infill line direction significantly affected the tensile strength of 3D printed samples, optimizing these parameters to enhance tensile strength while minimizing material consumption. Tamașag et al. [21] indicated that infill density had the greatest impact on the tensile strength, followed by the material, color pigment, and coloring percentage.

Heidari et al. [22] examined the impact of three important process parameters on the tensile properties of PLA samples. Their experimental results indicated that the optimal parameters for Young’s modulus and ultimate tensile strength were an infill density of 80%, print speed of 40 mm/s, and layer height of 0.1 mm. Rasheed et al. [23] found that infill density was the most influential parameter, while the hot bed temperature had the least influence. Kam et al. [24] determined that layer height was the most effective factor for enhancing mechanical properties, with optimal values being a layer height of 0.25 mm, an infill rate of 50%, a rectangular infill structure, and an extrusion temperature of 250 °C. Hikmat et al. [25] showed that construction direction, nozzle diameter, and infill density significantly affected part strength, with construction direction having the greatest impact on tensile strength. Othmani et al. [26] found that the maximum ultimate tensile strength could be achieved with an infill density of 30% and a concentric circle pattern, highlighting the importance of these factors. Mishra et al. [27] developed a mathematical equation capable of predicting the tensile strength of the selected material with a confidence level of 95%, showing that ABS and carbon fiber PLA met the expected tensile strength values under optimal conditions. Adibi et al. [28] reported that the tensile strength of ABS composite with 5% copper particles was 12.3% higher than that of the ABS material, with layer height and infill pattern being significant factors affecting the tensile strength of the printed material in the FDM process. Goutham et al. [29] identified that the maximum tensile strength parameters were achieved with an infill density of 50% and 20% recycled ABS.

PETG is a thermoplastic polymer material that is widely used in 3D printing. Due to its excellent mechanical properties and versatility, PETG has become a prominent choice following polylactic acid (PLA) and acrylonitrile butadiene styrene (ABS) [30]. PETG exhibits high strength and ductility, allowing it to withstand substantial physical stress and impact without breaking. Additionally, PETG offers good resistance to various chemicals (e.g., acids, alkalis, and solvents), making it suitable for applications that require chemical durability. Its transparency is another notable feature, making PETG an ideal material for 3D printing projects needing a transparent or translucent effect [31]. The following discusses the parameters for optimizing the tensile strength of PETG in FDM, as reported in previous studies.

Bembenek et al. [32] found that increasing print temperature and infill percentage improved the specific tensile strength of PETG and PLA parts, though it reduced the strength-to-weight ratio. Durgashyam et al. [33] indicated that layer height had a greater impact than feed rate and infill density. Layer height contributed 57.82% to tensile strength and 41.87% to flexural strength, demonstrating that PETG exhibits good mechanical properties with smaller layer heights. Panneerselvam et al. [34] showed that with process parameters of an infill density of 80%, a layer height of 0.3 mm, and a hexagonal infill pattern, PETG demonstrated enhanced mechanical properties. Srinivasan et al. [35] found that with a layer height of 0.1 mm and an infill density of 80%, the grid infill type achieved higher tensile strength compared to the triangle infill type, which had lower tensile strength due to continuous print contacts. Guessasma et al. [36] noted that FDM processing significantly degrades the mechanical properties, particularly the elongation at break. This degradation is attributed to the uneven deformation of the PETG during processing. Therefore, while FDM processing significantly reduces the elongation, rigidity, and tensile strength of PETG, it can also improve its fracture toughness.

As presented in Table 1, this paper compiles the optimization of the tensile strength for FDM, including research methods, materials, experimental parameters, and the most influential factors in tensile strength. A literature review revealed that PLA is the most commonly studied research material, followed by ABS, both of which have been used in 3D printing for several years. Due to the extensive research on these materials, the relatively newer PETG, which represents the third most common material, has been selected for this study to enhance its contribution to the field.

This study selects the infill density, layer height, infill line direction, and print temperature as control factors based on their significant influence on the FDM parameters. The infill line direction interacts with the construction direction and infill type. To maintain consistency in the infill line direction, the construction direction and infill type are excluded from the control factors. The construction direction is fixed with the largest plane as the datum plane parallel to the printing platform, and a straight line is used as the infill type for slicing and printing. Additionally, this study incorporates print speed as a parameter based on popular FDM parameters identified in previous research.

## 3. Research Method and Process

In this experiment, the tensile test model was created according to the ASTM D638-14 standard, designed as a 3D model in SOLIDWORKS 2022 (Paris, France. Dassault Systèmes), and then converted into an STL file. The model was then processed using PING SLICER 2.1 (Taichung City, Taiwan. Linkin Factory Co., Ltd.), where it was sliced parallel to the printing plane with specified layer heights, and post-processed into .gcode format for reading by an P200 FDM printer (Taichung City, Taiwan. Linkin Factory Co., Ltd.) to produce the specimen. The tensile test was conducted using a UH-100C (Kyoto, Japan. SHIMADZU CORPORATION) universal tensile testing machine, and the resulting experimental data were analyzed in Minitab Statistical Software 22 (Pennsylvania State College, USA), employing the Taguchi method and analysis of variance (ANOVA).

**Figure 1:**
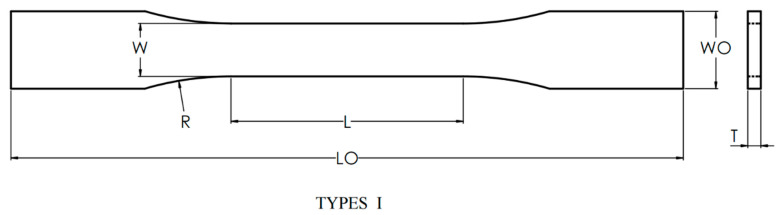
ASTM D638-14 Type I [37].

**Figure 2:**
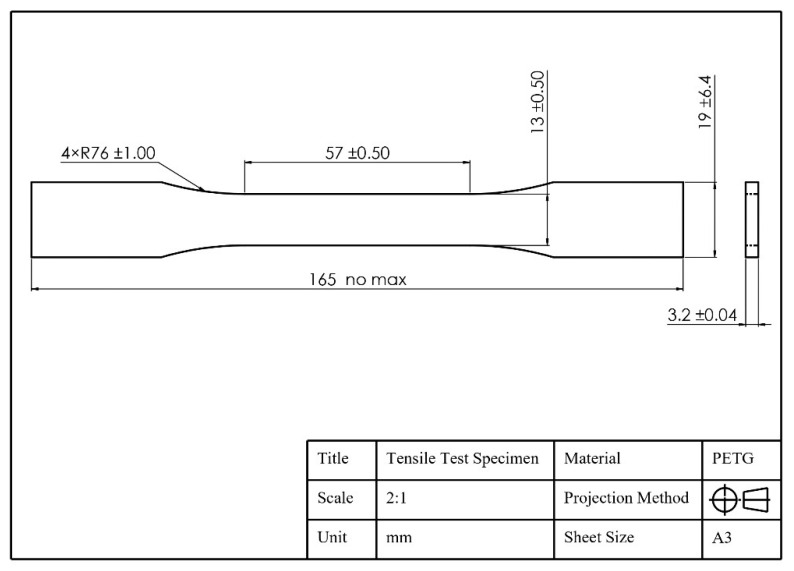
ASTM D638-14 Type I engineering drawing.

The printing material used for the test model in this paper is PETG, produced by SpiderMaker. (Taoyuan City, Taiwan), a modified polyester produced by introducing CHDM into PET. In this study, PETG is referred to simply as PETG. The tensile test model follows ASTM D638-14: Standard Test Method for Tensile Properties of Plastics [37]. According to the specifications, specifically for the sixth item, tensile samples of rigid or semi-rigid plastics should use the Type I sample, as depicted in Figure 1. The eighth item defines that the Test Speed must be the lowest at which the Type I sample can break within 0.5 to 5 min. Therefore, the tensile test in this study was conducted at a speed of 5 mm/min. The tensile samples are printed according to the dimensions shown in Figure 2 to facilitate data analysis and determine the maximum tensile strength. 

To obtain the maximum tensile strength and maximum unit cost-effectiveness, the Taguchi method employed in this paper uses a “larger-the-better” quality characteristic. Unit cost-effectiveness is defined as the tensile strength ratio relative to both the sample weight and print time. Thus, the cost comprises the materials and time required for printing, with units expressed as MPa/g (tensile strength per unit weight) and MPa/s (tensile strength per unit time). Two major conclusions are drawn from the results.

The experimental factors and their variation levels derived from the literature are summarized in Table 1. Based on this, the five quality control factors for this experiment are defined as layer height, infill density, print temperature, print speed, and infill line direction.

The variation ranges for these factors, informed by the results of Hikmat [25], Durgashyam [33], Panneerselva [34], Mardlotila [38], Mallikarjuna [39], Yadav [40], and Ajay et al. [41], are as follows:

(1) A: layer height 0.05 mm, 0.15 mm, 0.20 mm, and 0.30 mm.
(2) B: infill density 20%, 40%, 60%, and 80%.
(3) C: print temperature 220 °C, 230 °C, 240 °C, and 250 °C.
(4) D: print speed 30 mm/s, 40 mm/s, 50 mm/s, and 60 mm/s.
(5) E: infill line direction: 0°, 30°, 60°, and 90°.

**Figure 3:**
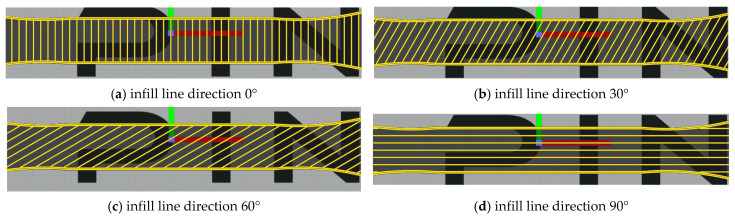
Infill line direction schematic diagram.

In this study, the infill line direction, an additional quality control factor, is defined by the rotation angle of the *Y*-axis (green axis) of the tensile sample model relative to the *X*-axis (red axis). Specifically, 0° corresponds to the *Y*-axis (green axis) and 90° corresponds to the *X*-axis (red axis), the yellow color is the printing path of the nozzle.as illustrated in Figure 3.

The orthogonal array (OA) employed in this paper is $L_{16}$, which is used to test the reproducibility of the factor effects. The Signal-to-Noise (S/N) ratio calculation formula is given by Equation (1):

(1) $${S/N}_{LB}=-10log\left(\frac{1}{n}\sum_{i=1}^{n}\frac{1}{y_{i}^{2}}\right)$$

Equation (2) is used to predict and confirm the confidence intervals of the values, which serves as the basis for evaluating the accuracy of the experimental data and experimental models.

(2) $$CI=\pm \sqrt{\frac{S^{2}}{m_{e}}+\frac{S^{2}}{m_{r}}}\times TINV\left(1-\alpha \%,dof\right)$$

Finally, ANOVA is utilized to examine the calculated values of each control factor through various statistical analyses, resulting in preliminary and secondary variation analysis tables, which are then analyzed and compared.

## 4. Results and Discussion

### 4.1. Taguchi Experimental Method

The tensile tests were conducted according to $L_{16}$ OA and Table 2, including the maximum tensile strength. The maximum tensile strength per unit weight and maximum tensile strength per unit time are summarized in Table 3.

Based on the tensile test results (Table 3), parameter combination No. 4 yields the highest tensile strength and S/N. This combination includes a layer height of 0.05 mm, infill density of 80%, print temperature of 250 °C, print speed of 60 mm/s, and infill line direction of 90°. This combination results in a tensile strength of 40.32 MPa and S/N ratio of 32.11 dB.

For the tensile strength per unit weight, the same No. 4 parameter combination also provides the highest tensile strength per unit weight and S/N. The tensile strength per unit weight is 4.03 MPa/g, with an S/N ratio of 12.11 dB, achieved with a layer height of 0.05 mm, an infill density of 80%, a print temperature of 250 °C, a print speed of 60 mm/s, and an infill line direction of 90°.

For tensile strength per unit time, parameter combination No. 15 demonstrates the highest tensile strength per unit time and S/N ratio. This combination includes a layer height of 0.30 mm, an infill density of 60%, a print temperature of 230 °C, a print speed of 60 mm/s, and an infill line direction of 0°. It results in a tensile strength per unit time of 79.39 MPa/min and an S/N ratio of 38.00 dB.

**Figure 4:**
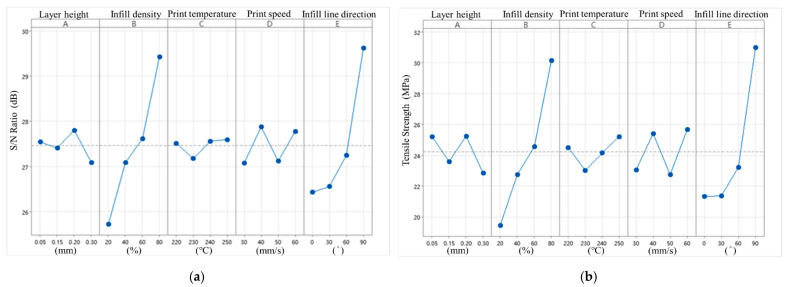
Tensile strength S/N ratio factor response diagram and quality characteristic factor response diagram. (**a**) S/N ratio factor response diagram (tensile strength). (**b**) Quality characteristic factor response diagram (tensile strength).

According to the Delta values and ranking presented in Table 4 and Figure 4, the infill density has the greatest impact on the tensile strength among the five control factors in the S/N ratio analysis, with a Delta value of 3.70. In contrast, the print temperature has the least impact, with a Delta value of 0.42. The optimal parameter combination predicted based on the S/N ratio analysis is A3B4C4D2E4.

In terms of quality characteristics, infill density remains the most influential factor, with a Delta value of 10.69, while print temperature has the least impact, with a Delta value of 2.20. This finding aligns with the S/N ratio analysis trend. The order of importance for the control factors, ranked from most to least significant, is A-E: 41532. However, the optimal parameter combination predicted for the quality characteristics differs from the S/N ratio analysis, with the updated values being A3B4C4D4E4. Notably, the print speed is increased from 40 mm/s to 60 mm/s compared to the S/N ratio analysis.

**Figure 5:**
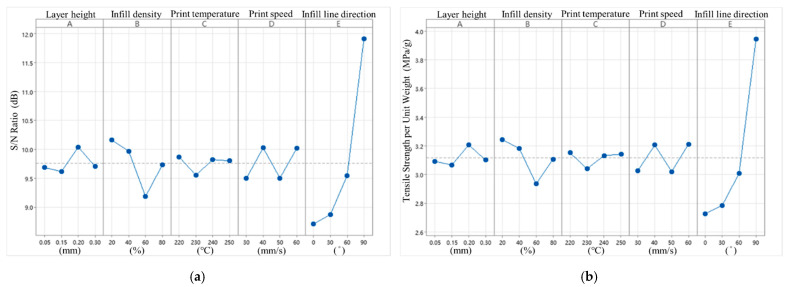
Tensile strength per unit weight S/N ratio factor response diagram and quality characteristic factor response diagram. (**a**) S/N ratio factor response diagram (tensile strength per unit weight). (**b**) Quality characteristic factor response diagram (tensile strength per unit weight).

According to the Delta values and ranking presented in Table 5 and Figure 5, the infill line direction has the greatest impact on the tensile strength per unit weight in the S/N ratio analysis, with a Delta value of 3.21. In contrast, the print temperature has the least impact, with a Delta value of 0.31. Therefore, the infill density becomes less significant when focusing on maximizing the tensile strength per unit weight. Since high infill density contributes to increased weight, optimizing for MPa/g (tensile strength per unit weight) prioritizes other factors. The infill line direction emerges as a more critical factor in this context. The predicted optimal parameter combination is A3B1C1D2E4.

Among the five control factors for quality characteristics, the infill line direction has the greatest impact on the tensile strength per unit weight, with a Delta value of 1.218. In contrast, the print temperature still has the least impact, with a Delta value of 0.112. This finding is consistent with the trend observed in the S/N ratio analysis. The optimal parameter combination for maximizing the tensile strength per unit weight is A3B1C1D4E4. Due to the minimal difference between variation levels 2 and 4 for print speed (D) in Table 5, the effective digits in this table are rounded to three decimal places to ensure precision.

**Figure 6:**
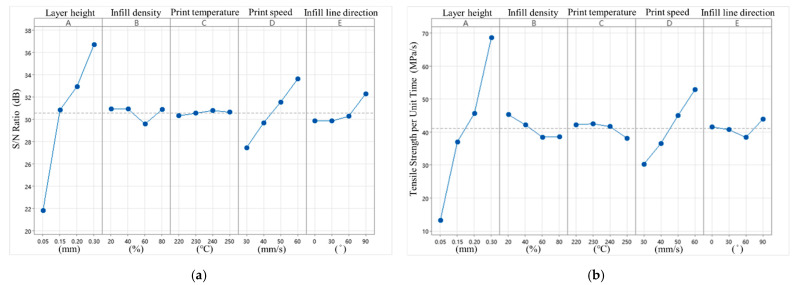
Tensile strength per unit time S/N ratio factor response diagram and quality characteristic factor response diagram. (**a**) S/N ratio factor response diagram (tensile strength per unit time). (**b**) Quality characteristic factor response diagram (tensile strength per unit time).

According to the Delta values and ranking in Table 6 and Figure 6, the layer height of S/N has the greatest impact on the tensile strength per unit time, with a Delta value of 14.87. In contrast, the print temperature has the least impact, with a Delta value of 0.47. Therefore, the infill density and infill line direction become less significant when considering the tensile strength per unit time. Since a smaller layer height results in more layers and longer print times, and the goal is to maximize MPa/min, the layer height will replace both. The predicted optimal parameters are A4B2C3D4E4.

Among the five control factors for quality characteristics, layer height still has the greatest impact on tensile strength per unit time, with a Delta value of 55.35. In contrast, the print temperature still has the least impact, with a Delta value of 4.33. This finding aligns with the trend of the S/N ratio analysis. However, the infill line direction has a greater impact than the infill density in this context. The predicted optimal parameters are A4B1C2D4E4.

### 4.2. Analysis of Variance (ANOVA)

According to the analysis results in Table 7, the primary contributing factors to the tensile strength are the infill density (48.45%) and infill line direction (45.26%). This indicates that variations in these two factors have the greatest impact on the tensile strength. Conversely, the print temperature contributes minimally, with a contribution factor of only 0.78%, making it the least influential factor on the tensile strength. To optimize tensile strength effectively, focusing on adjusting the infill density and infill line direction is crucial. Therefore, the print temperature should be considered as part of the experimental errors, and ANOVA should be conducted again to obtain accurate statistics for the key control factors.

Table 8 shows the significant influencing factors, which are the infill density and infill line direction. These factors substantially impact the tensile strength, leading to the rejection of the null hypothesis for these variables. In contrast, the layer height and print speed are identified as non-significant influencing factors, as they do not substantially impact the tensile strength. Thus, the null hypothesis for these factors is not rejected.

According to the analysis results in Table 9, the primary contributing factor to the tensile strength is the infill line direction (87.22%). This indicates that variations in this factor have the greatest contribution to the tensile strength per unit weight. Conversely, print temperature contributes minimally, with a factor contribution of only 0.77%. To optimize tensile strength per unit weight effectively, focus should be placed on adjusting the infill line direction. Therefore, the print temperature should be considered part of the experimental errors, and ANOVA should be conducted again to obtain accurate statistics for the key control factors.

Table 10 shows that the infill line direction is the only significant factor influencing the tensile strength per unit weight. This factor has a substantial impact, leading to the rejection of the null hypothesis for this variable. In contrast, layer height, infill density, and print speed are identified as non-significant influencing factors for tensile strength per unit weight, and the null hypothesis for these factors is not rejected.

According to the analysis results in Table 11, the main contributing factor to the tensile strength per unit time is the layer height (82.12%). This indicates that variations in the layer height have the greatest contribution to the tensile strength per unit time. In contrast, print temperature contributes minimally, with a factor contribution of only 0.08%. Thus, optimizing the tensile strength per unit time should focus on adjusting the layer height. Therefore, print temperature should be included in the experimental errors, and ANOVA should be used again to obtain accurate statistics for the key control factors.

Table 12 shows that the significant factors influencing the tensile strength per unit time are the layer height, print speed, and infill line direction. These factors have a significant impact, leading to the rejection of their null hypotheses. In contrast, the infill density does not significantly impact the tensile strength per unit time, and the null hypothesis for this factor is not rejected.

### 4.3. Investigation of Tensile Testing

**Figure 7:**
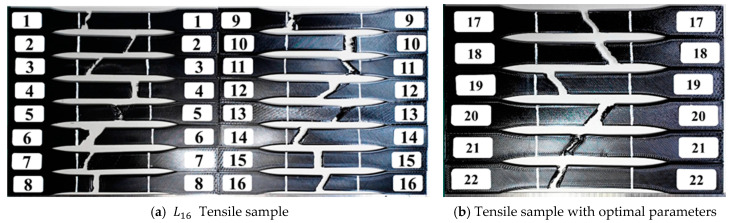
Tensile sample breaking.

Figure 7a illustrates that the cross-section angle of each tensile sample closely matches the angles of the infill line direction. Specifically, samples Nos. 1, 4, 5, 8, 10, and 15 have breaking angles approximately normal to the *X*-axis, with their infill line directions being 0° or 90°. This suggests that the internal infill structure influences the tensile stress distribution. The samples tend to break along the infill line direction when they cannot break perpendicularly.

Figure 7b presents the arrangement of the tensile samples based on three quality characteristics: tensile strength, tensile strength per unit weight, and tensile strength per unit time. The samples with the optimal S/N ratio and quality characteristics are Nos. 17, 18, 19, 20, 21, and 22. Although the infill line direction of these samples is 90°, this does not fully explain their behavior. It is observed that when the cross-section of these samples attempts to align perpendicular to the *X*-axis, the bottom and top layers’ print patterns constrain this alignment, resulting in a slightly vertical orientation at the ends and a noticeable print angle in the middle part.

Next, we analyze the tensile strength quality characteristics by examining the advantages and disadvantages of the physical properties. According to Table 13, Sample 17 demonstrates a higher maximum tensile stress and load capacity compared to Sample 4, indicating superior tensile strength and confirming the effectiveness of the optimal parameters. By increasing the layer height and reducing the printing speed, we achieved an additional 1.819 MPa maximum tensile strength and an elastic modulus of 21.369. These results are the outcomes of optimizing the control factors using the Taguchi method, and an assessment of each control factor is presented below.

First, reducing the printing speed allows for more complete bonding between the PETG molecular layers in FDM, as a slower extrusion rate decreases the cooling curve slope, reducing the internal stress and enhancing the overall structural integrity.

Increasing the layer height, on the other hand, implies the extrusion of more material, improving material flow, and increasing the contact area between layers, leading to higher interlayer fusion. This phenomenon likely contributes to minimizing internal defects and voids within the sample, resulting in superior performance.

### 4.4. Verification of Optimal Parameters

Based on the Taguchi method analysis, the optimal parameters for the tensile strength are A3B4C4D2E4 for the S/N ratio and A3B4C4D4E4 for the quality characteristics. The only difference between these sets of parameters is the print speed. Since the influence of print temperature has been pooled into experimental errors in the secondary ANOVA, its effect is not considered in the final analysis. The confidence interval is calculated using Equation (2) and compared with the verification experiment values in Table 14. According to Table 14, the parameter combination based on the S/N ratio is more suitable for optimizing the tensile strength. Compared to the maximum values observed in the orthogonal array (S/N ratio of 32.11 dB and tensile strength of 40.32 MPa), the S/N ratio increased by 0.38 dB, and the tensile strength increased by 1.82 MPa.

The optimal tensile strength per unit weight of the S/N ratio is A3B1C1D2E4, while the parameters for optimal tensile strength per unit weight of quality characteristics are A3B1C1D4E4. The only difference between them is the print speed. According to Table 14, the parameter combination based on the S/N ratio is more suitable for optimizing the tensile strength per unit weight. Compared to the maximum values in the orthogonal array (S/N ratio of 12.11 dB and tensile strength per unit weight of 4.03 MPa/g), the S/N ratio increased by 0.31 dB, and the tensile strength per unit weight increased by 0.15 MPa/g.

The optimal tensile strength per unit weight of the S/N ratio is A4B2C3D4E4, while the optimal tensile strength per unit weight of the quality characteristics is A4B1C2D4E4. Here, the differences lie in the infill density and print temperature. According to Table 14, the parameter combination based on the S/N ratio is more suitable for optimizing the tensile strength per unit weight. Compared to the maximum values in the orthogonal array (S/N ratio of 12.11 dB and tensile strength per unit weight of 4.03 MPa/g), the S/N ratio increased by 0.31 dB, and the tensile strength per unit weight increased by 0.15 MPa/g.

### 4.5. Experiment Summary

The most influential factors for the three quality characteristics are summarized in Table 15. The infill density should be considered the primary factor affecting the tensile strength of the printed sample. This conclusion aligns with the findings of previous studies [18,19,21,22,23,32,34].

For the tensile strength per unit weight, the direction of the infill line is the most critical factor. This indicates that the orientation of the infill line is more important than the amount of infill used. To achieve the maximum tensile strength while using the minimal infill density, the infill line direction should be the primary consideration.

Regarding the tensile strength per unit time, the focus should be on the layer height. Regardless of the chosen layer height, the time required for each layer remains consistent. Thus, selecting a larger layer height is advantageous for maintaining the tensile strength while improving the printing efficiency.

Lastly, print temperature is the least influential factor among the three quality characteristics, with its impact on each quality characteristic being less than 1%. Its effect on the tensile strength per unit time is below 0.1%. This minimal influence may result from insufficient variation in print temperature levels. However, it is difficult to form a tensile sample beyond this range, and a tensile test cannot be performed. Therefore, print temperatures between 220 °C and 250 °C are suitable for all three quality characteristics, and reducing the temperature within this range can help lower printing costs.

In addition, the average values from the 16 experiments constructed for the three quality characteristics are compared and summarized in Table 16. The S/N formula (Equation (1)) calculates the quality loss and standard deviation between the optimal and average values (Equations (3) and (4)). The equations are presented in a straightforward manner, avoiding complex mathematical symbols to ensure clarity.

(3) $$quality\;loss=0.5^{\frac{optimal-average}{3}}$$

(4) $$standard\;deviation=0.5^{\frac{optimal-average}{6}}$$

Table 16 confirms that the verified experimental values for each quality characteristic fall within the confidence intervals, demonstrating that constructing the three experimental models is reliable and accurate. The experimental data are evaluated with a 99% confidence interval.

For the tensile strength, using the optimal parameter values (A3B4C4D2E4) derived from Taguchi and ANOVA, the average quality loss can be reduced to 31.28%, and the standard deviation can be reduced to 55.93%. For the tensile strength per unit weight, by applying the optimal parameters (A3B1C1D2E4), the original Taguchi experiment average quality loss can be reduced to 54.09%, and the standard deviation can be reduced to 73.54%. For the tensile strength per unit time, using the optimal parameter values of Taguchi and ANOVA (A4B2C3D4E4) from Taguchi and ANOVA results in a reduction of the average quality loss by 10.81% and a reduction in the standard deviation by 32.87%.

The above analysis and experimental data indicate that the performance of each quality characteristic can be significantly improved through the Taguchi experimental design and ANOVA, resulting in reduced quality loss and standard deviation. The verification experiment values falling within the confidence intervals further confirm the reliability and accuracy of the experimental models. These results are crucial for enhancing the quality control of FDM parameters and provide a practical reference for future experiments and product development.

## 5. Conclusions

This paper investigates the effects of various 3D printing parameters on the tensile strength, tensile strength per unit weight, and tensile strength per unit time of PETG material using FDM. The results from the Taguchi experiments and ANOVA analysis are summarized as follows:

1. Tensile strength: The infill density has the greatest impact, contributing 48.45% to the variation, whereas the print temperature has the least impact, contributing only 0.78%. The optimal parameter combination (A3B4C4D2E4) reduces the quality loss by 31.28% and the standard deviation by 55.93%. The recommended parameters for maximum tensile strength are a layer height of 0.20 mm, infill density of 80%, print temperature of 250 °C, print speed of 40 mm/s, and infill line direction of 90°.
2. Tensile strength per unit weight: The infill line direction has the greatest impact, accounting for 87.22% of the variation, while the print temperature has the least impact, which is only 0.77%. The optimal parameter combination (A3B1C1D2E4) reduces the quality loss by 54.09% and the standard deviation by 73.54%. The optimal parameters for the maximum tensile strength per unit weight are a layer height of 0.20 mm, an infill density of 20%, a print temperature of 220 °C, a print speed of 40 mm/s, and an infill line direction of 90°.
3. Tensile strength per unit time: The layer height has the greatest impact, accounting for 82.12%, while the print temperature has the least impact, contributing only 0.08%. The optimal parameter combination (A4B2C3D4E4) reduces the quality loss by 10.81% and the standard deviation by 32.87%. The optimal parameters for the maximum tensile strength per unit time are a layer height of 0.30 mm, an infill density of 40%, a print temperature of 240 °C, a print speed of 60 mm/s, and an infill line direction of 90°.

The findings from this study offer crucial insights for optimizing the FDM parameters for PETG materials. Based on the application in engineering practice, suggestions for printing parameters are provided for two major aspects. To achieve the best performance of the printed model in terms of tensile strength, the recommended parameters are a layer height of 0.20 mm, an infill density of 80%, a print temperature of 250 °C, a print speed of 40 mm/s, and an infill line direction of 90°. For the highest unit cost-effectiveness material usage, energy consumption, and time, the suggested FDM parameters are a layer height of 0.30 mm, an infill density of 20%, a print temperature of 220 °C, a print speed of 60 mm/s, and an infill line direction of 90°.

## Figures and Tables

**Table 1:** Literature summary.

First Author	Research Method	Material	FDM Parameter	Greatest Influencing Factor
Agrawal, Anant Prakash [16]	Taguchi method ($L_{9}$)	ABS	Infill type Infill density Layer height	Infill type
Ahmad, Mohd Nazri [17]	Taguchi method ($L_{9}$) ANOVA	ABS and oil palm fiber composite	Infill density Infill line direction Print speed Layer height	Infill line direction
Fountas, N. A. [18]	Taguchi method ($L_{16}$) Response Surface Method Linear Regression	PLA	Shell thickness Layer height Infill density Construction direction Print speed	Infill density
Kam, M. [19]	Taguchi method ($L_{8}$) ANOVA	PLA+fiber material	Infill type Infill density Construction direction	Infill density
Korkut, Volkan [20]	Taguchi method ($L_{25}$) ANOVA Linear Regression	PLA	Line width Number of shell lines Infill line direction Infill overlap area	Infill overlap area
Tamașag, Ioan [21]	Taguchi method ($L_{27}$) ANOVA	PLA PLA+ PETG	Infill density Material type Color type Coloring percentage	Infill density
Heidari-Rarani, M. [22]	Taguchi method ($L_{8}$)	PLA	Layer height Print speed Infill density	Infill density
Rasheed, Adnan [23]	Taguchi method ($L_{9}$)	PLA-ABS double layer composite	Infill density Number of layers Print speed Print temperature	Infill density
Kam, Menderes [24]	Taguchi method ($L_{8}$) ANOVA Linear Regression	PA 12	Layer height Infill density Infill type Print temperature	Layer height
Hikmat, Mohammed [25]	Taguchi method ($L_{8}$) ANOVA Linear Regression	PLA	Construction direction Infill line direction Nozzle diameter Print temperature Infill density Number of shells Print speed	Construction direction
Othmani, M. [26]	Taguchi method ($L_{16}$) ANOVA	ABS	Infill pattern Infill density	Infill pattern
Mishra, Debashis [27]	Taguchi method ($L_{16}$) Linear Regression	ABS Carbon fiber PLA	Print speed Infill density Layer height	N/A
Adibi, Hamed [28]	Taguchi method ($L_{9}$) ANOVA	Copper-ABS composite	Print temperature Infill pattern Layer height	Layer height
Goutham, R. [29]	Taguchi method ($L_{8}$) ANOVA	Recycled ABS	Material type Infill density	Material type
Bembenek, Michał [32]	Fractional Factorial Design	PLA PETG	Print temperature Infill type Infill density Layer height Construction direction	Infill density
Durgashyam, K. [33]	ANOVA Linear Regression	PETG	Layer height Print speed Infill density	Layer height
Panneerselvam, T. [34]	Taguchi method ($L_{8}$)	PETG	Infill density Layer height Infill type	Infill density
Srinivasan, R. [35]	Full Factorial Method	PETG	Layer height Infill type Infill density	N/A
Guessasma, Sofiane [36]	Microscanning Mechanical property test Finite element analysis	PETG	Print temperature Baseplate temperature Material type	Print temperature

Note. N/A = Not Available.

**Table 2:** Control factors and change levels.

Experimental Factors	Experimental Level			
	1	2	3	4
A. Layer height (mm)	0.05	0.15	0.20	0.30
B. Infill density (%)	20	40	60	80
C. Print temperature (°C)	220	230	240	250
D. Print speed (mm/s)	30	40	50	60
E. Infill line direction (°)	0	30	60	90

**Table 3:** Data and S/N values of each group of the Taguchi experiment (three types of quality characteristics).

No.	Parameters					Tensile Strength (MPa)	S/N Ratio (dB)	Tensile Strength per Unit Weight (MPa/g)	S/N Ratio (dB)	Tensile Strength per Unit Time (MPa/s)	S/N Ratio (dB)
	A	B	C	D	E						
1	0.05	20	220	30	0	16.66	24.43	2.78	8.87	8.03	18.09
2	0.05	40	230	40	30	20.86	26.39	2.84	9.06	10.61	20.51
3	0.05	60	240	50	60	22.99	27.23	2.72	8.69	12.15	21.69
4	0.05	80	250	60	90	40.32	32.11	4.03	12.11	22.20	26.93
5	0.15	20	230	50	90	22.90	27.20	3.85	11.71	49.08	33.82
6	0.15	40	220	60	60	22.86	27.18	3.15	9.97	48.12	33.65
7	0.15	60	250	30	30	20.91	26.41	2.49	7.92	20.07	26.05
8	0.15	80	240	40	0	27.72	28.86	2.77	8.86	30.80	29.77
9	0.20	20	240	60	30	18.99	25.57	3.11	9.87	61.60	35.79
10	0.20	40	250	50	0	20.38	26.18	2.81	8.98	47.95	33.62
11	0.20	60	220	40	90	33.81	30.58	3.98	11.99	42.26	32.52
12	0.20	80	230	30	60	27.78	28.88	2.92	9.32	30.87	29.79
13	0.30	20	250	40	60	19.24	25.68	3.23	10.19	62.41	35.90
14	0.30	40	240	30	90	26.86	28.58	3.92	11.87	61.99	35.85
15	0.30	60	230	60	0	20.51	26.24	2.55	8.12	79.39	38.00
16	0.30	80	220	50	30	24.71	27.86	2.70	8.63	70.60	36.98
Average						24.22	27.46	3.12	9.76	41.13	30.56

**Table 4:** Factor response table (tensile strength).

	S/N Ratio					Quality Characteristic				
Factor Level	(A)	(B)	(C)	(D)	(E)	(A)	(B)	(C)	(D)	(E)
Level 1.	27.54	25.72	27.51	27.07	26.43	25.21	19.45	24.51	23.05	21.32
Level 2.	27.41	27.08	27.17	27.88	26.56	23.60	22.74	23.01	25.41	21.37
Level 3.	27.80	27.61	27.56	27.12	27.24	25.24	24.56	24.14	22.75	23.22
Level 4.	27.09	29.43	27.60	27.78	29.62	22.83	30.14	25.21	25.67	30.97
Effect	0.71	3.70	0.42	0.80	3.19	2.41	10.69	2.20	2.93	9.66
Rank	4	1	5	3	2	4	1	5	3	2

**Table 5:** Factor response table (tensile strength per unit weight).

	S/N Ratio					Quality Characteristic				
Factor Level	(A)	(B)	(C)	(D)	(E)	(A)	(B)	(C)	(D)	(E)
Level 1.	9.68	10.16	9.87	9.50	8.71	3.092	3.243	3.152	3.028	2.727
Level 2.	9.62	9.97	9.55	10.03	8.87	3.066	3.181	3.040	3.206	2.785
Level 3.	10.04	9.18	9.82	9.50	9.55	3.207	2.934	3.132	3.020	3.008
Level 4.	9.70	9.73	9.80	10.02	11.92	3.101	3.107	3.142	3.212	3.945
Effect	0.42	0.98	0.31	0.53	3.21	0.141	0.309	0.112	0.191	1.218
Rank	4	2	5	3	1	4	2	5	3	1

**Table 6:** Factor response table (tensile strength per unit time).

	S/N Ratio					Quality Characteristic				
Factor Level	(A)	(B)	(C)	(D)	(E)	(A)	(B)	(C)	(D)	(E)
Level 1.	21.81	30.90	30.31	27.45	29.87	13.25	45.28	42.25	30.24	41.54
Level 2.	30.82	30.91	30.53	29.68	29.83	37.02	42.17	42.49	36.52	40.72
Level 3.	32.93	29.57	30.78	31.53	30.26	45.67	38.47	41.64	44.94	38.39
Level 4.	36.68	30.87	30.62	33.59	32.28	68.60	38.62	38.16	52.83	43.88
Effect	14.87	1.34	0.47	6.14	2.44	55.35	6.81	4.33	22.59	5.49
Rank	1	4	5	2	3	1	3	5	2	4

**Table 7:** Primary ANOVA (tensile strength).

Factor	*SS*	*DOF*	*Var*	*ρ*
A. Layer height (mm)	1.05	3	0.35	1.81%
B. Infill density (%)	28.2	3	9.4	48.45%
C. Print temperature (°C)	0.45	3	0.15	0.78%
D. Print speed (mm/s)	2.16	3	0.72	3.71%
E. Infill line direction (°)	26.34	3	8.78	45.26%
Total	58.2	15	19.4	100%

**Table 8:** Secondary ANOVA (tensile strength).

Factor	*SS*	*DOF*	*Var*	*F*	*ρ*	Confidence	Significant?
A. Layer height (mm)	1.05	3	0.35	2.33	1.81%	74.71%	NO
B. Infill density (%)	28.2	3	9.4	62.41	48.45%	99.67%	YES
C. Print temperature (°C)	pooled						
D. Print speed (mm/s)	2.16	3	0.72	4.78	3.71%	88.44%	NO
E. Infill line direction (°)	26.34	3	8.78	58.3	45.26%	99.63%	YES
Error	0.45	3	0.15	S = 0.39			
Total	58.2	15	19.4	Note: At least 99% Confidence			

**Table 9:** Primary ANOVA (tensile strength per unit weight).

Factor	*SS*	*DOF*	*Var*	*ρ*
A. Layer height (mm)	0.42	3	0.14	1.39%
B. Infill density (%)	2.13	3	0.71	7.02%
C. Print temperature (°C)	0.23	3	0.08	0.77%
D. Print speed (mm/s)	1.09	3	0.36	3.59%
E. Infill line direction (°)	26.46	3	8.82	87.22%
Total	30.33	15	10.11	100%

**Table 10:** Secondary ANOVA (tensile strength per unit weight).

Factor	*SS*	*DOF*	*Var*	*F*	*ρ*	Confidence	Significant?
A. Layer height (mm)	0.42	3	0.14	1.8	1.39%	67.88%	NO
B. Infill density (%)	2.13	3	0.71	9.06	7.02%	94.84%	NO
C. Print temperature (°C)	pooled						
D. Print speed (mm/s)	1.09	3	0.36	4.64	3.59%	88.02%	NO
E. Infill line direction (°)	26.46	3	8.82	112.58	87.22%	99.86%	YES
Error	0.23	3	0.08	S = 0.28			
Total	30.33	15	10.11	Note: At least 99% Confidence			

**Table 11:** Primary ANOVA (tensile strength per unit weight).

Factor	*SS*	*DOF*	*Var*	*ρ*
A. Layer height (mm)	479.05	3	159.68	82.12%
B. Infill density (%)	5.29	3	1.76	0.91%
C. Print temperature (°C)	0.46	3	0.15	0.08%
D. Print speed (mm/s)	82.37	3	27.46	14.12%
E. Infill line direction (°)	16.19	3	5.4	2.77%
Total	583.36	15	194.45	100%

**Table 12:** Secondary ANOVA (tensile strength per unit weight).

Factor	*SS*	*DOF*	*Var*	*F*	*ρ*	Confidence	Significant?
A. Layer height (mm)	479.05	3	159.68	1046.32	82.12%	99.99%	YES
B. Infill density (%)	5.29	3	1.76	11.55	0.91%	96.28%	NO
C. Print temperature (°C)	pooled						
D. Print speed (mm/s)	82.37	3	27.46	179.91	14.12%	99.93%	YES
E. Infill line direction (°)	16.19	3	5.4	35.35	2.77%	99.23%	YES
Error	0.46	3	0.15	S = 0.39			
Total	583.36	15	194.45	Note: At least 99% Confidence			

**Table 13:** Comparison Table of Tensile Data for Samples 4 and 17.

NO.	Print Parameter Configuration	Maximum Load (kN)	Maximum Tensile Stress (MPa)	Elastic Coefficient (MPa)
4	A1B4C4D4E4	1.678	40.325	472.068
17	A3B4C4D2E4	1.753	42.144	493.437

**Table 14:** Verification experiment values comparison table (tensile strength).

	Parameter Configuration	S/N Ratio	Quality Characteristic	Confidence Interval	Prediction Difference	Prediction Error
Tensile strength	A3B4C4D2E4	32.49	42.14	32.34 ± 3.05	0.15	0.4%
	A3B4C4D4E4	31.96	39.62	32.24 ± 3.05	0.28	0.8%
Tensile strength per unit weight	A3B1C1D2E4	12.42	4.18	12.86 ± 2.20	0.44	3.5%
	A3B1C1D4E4	11.75	3.87	12.85 ± 2.20	1.10	9.3%
Tensile strength per unit time	A4B2C3D4E4	40.19	102.25	41.78 ± 3.07	1.59	3.9%
	A4B1C2D4E4	40.06	100.70	41.77 ± 3.07	1.71	4.2%

**Table 15:** Greatest influencing factors for each quality characteristic.

Quality Characteristic	Greatest Influencing Factor, Influence (%)	Least Influencing Factor, Influence (%)
Tensile strength	Infill density (48.45%)	Print temperature, (0.78%)
Tensile strength per unit weight	Infill line direction (87.22%)	Print temperature, (0.77%)
Tensile strength per unit of time	Layer height (82.12%)	Print temperature, (0.08%)

**Table 16:** Experimental model construction effects.

Description	Tensile Strength	Tensile Strength per Unit Weight	Tensile Strength per Unit of Time
$L_{16}$ Average value of the Taguchi experiment	27.46 dB	9.76 dB	30.56 dB
Optimal value of Taguchi ANOVA	32.49 dB	12.42 dB	40.19 dB
Confidence interval of verification experiment values	[30.07, 34.61]	[11.23, 14.49]	[39.50, 44.06]
Quality loss reduction percentage	31.28%	54.09%	10.81%
Standard deviation reduction percentage	55.93%	73.54%	32.87%

## Data Availability

The original contributions presented in the study are included in the article, and further inquiries can be directed to the corresponding author/s.
